# Therapeutic Challenges of Post-traumatic Stress Disorder: Focus on the Dopaminergic System

**DOI:** 10.3389/fphar.2019.00404

**Published:** 2019-04-17

**Authors:** Sebastiano Alfio Torrisi, Gian Marco Leggio, Filippo Drago, Salvatore Salomone

**Affiliations:** Department of Biomedical and Biotechnological Sciences, University of Catania, Catania, Italy

**Keywords:** post-traumatic stress disorder, dopamine D_3_ receptor, intrusive symptoms, mood and cognition, arousal, SSRI

## Abstract

Post-traumatic stress disorder (PTSD) is a mental illness developed by vulnerable individuals exposed to life-threatening events. The pharmacological unresponsiveness displayed by the vast majority of PTSD patients has raised considerable interest in understanding the poorly known pathophysiological mechanisms underlying this disorder. Most studies in the field focused, so far, on noradrenergic mechanisms, because of their well-established role in either tuning arousal or in encoding emotional memories. However, less attention has been paid to other neural systems. Manipulations of the dopaminergic system alter behavioral responses to stressful situations and recent findings suggest that dopaminergic dysfunction might play an overriding role in the pathophysiology of PTSD. In the present review, dopaminergic mechanisms relevant for the pathogenesis of PTSD, as well as potential dopaminergic-based pharmacotherapies are discussed in the context of addressing the unmet medical need for new and effective drugs for treatment of PTSD.

## Introduction

Post-traumatic stress disorder (PTSD) is the most prevalent neuropsychiatric disorder developed by vulnerable individuals exposed to life-threatening events (Benjet et al., 2016). The probability of developing PTSD ranges from 5 to 31% according to different factors such as social background, home country and kind of traumatic event experienced (Girgenti et al., 2017; Shalev et al., 2017). The complex diagnosis of PTSD relies on several criteria described in the Diagnostic and Statistical Manual of Mental Disorders, fifth edition (DSM-5): an individual, after exposure to death, threatened death, actual or threatened serious injury, or actual or threatened sexual violence (Criterion A), must exhibit ongoing intrusive re-experiencing symptoms (Criterion B, one required), avoidance symptoms (Criterion C, one required), negative alterations in mood and cognitions (Criterion D, two required) and symptoms of hyperarousal (Criterion E, two required). These symptoms must last for more than 1 month (Criterion F) and cause a functional impairment (Criterion G). It is also important to exclude that these symptoms are triggered by medications or other psychiatric disorders (Criterion H). Besides the classic PTSD, a cluster of patients may experience a dissociative subtype of PTSD, characterized by additional symptoms of depersonalization and derealization (Fenster et al., 2018). The first-line pharmacotherapy for PTSD consists of two selective serotonin reuptake inhibitors (SSRI), namely paroxetine and sertraline, which are the only medications approved by the U.S. Food and Drug Administration. However, because among PTSD patients only a few (less than 30%) achieve full remission (Berger et al., 2009), there is an urgent need to discover and develop new effective medications. Several inconclusive approaches have been proposed to overcome this pharmacological unresponsiveness. The preventive therapy, which should be undertaken before the onset of the symptoms, indeed raises both economical and ethical issues, considering that some individuals are prone while others are resilient to develop PTSD after the trauma, and an univocal reliable method to identify the susceptible ones (those supposed to receive a preventive treatment) is still unavailable (Steckler and Risbrough, 2012). The results concerning the augmentation strategies with atypical antipsychotics for SSRI non-responders are also rather arguable (Krystal et al., 2011). One promising drug that could shed light on this issue is the non-competitive N-methyl-D-aspartate (NMDA) receptor antagonist ketamine. A key pilot randomized controlled trial demonstrated a rapid relief of PTSD symptoms after intravenous infusion of ketamine without the occurrence of dissociative symptoms (Feder et al., 2014). Despite these encouraging results, the efficacy and safety of repeated infusions of ketamine would need to be tested in further confirmatory clinical trials. Indeed, preclinical studies suggest that ketamine might promote the development of PTSD (Morena et al., 2017; Radford et al., 2018).

A promising, recently proposed, strategy is the combination of pharmacological treatment and cognitive behavioral therapy (CBT, Morena et al., 2018). In PTSD, CBT consists of repeated exposure of the patient to trauma-related reminders in a safe controlled situation. It is believed that this kind of exposure-based psychotherapy may trigger extinction learning processes, which could result effective in a subset of PTSD patients. However, a substantial number of PTSD patients drops out of CBT, while a high rate of relapse of PTSD symptoms has been observed after the end of CBT (Gutner et al., 2016). In this regard, either drugs able to counteract the excessive retrieval or drugs that can enhance the extinction of traumatic memories could increase the efficacy of CBT (Morena et al., 2018).

Increasing our understanding of the neurobiology of a given disease is essential for the development of new effective and safe medications (Everitt et al., 2018). The etiology and the pathophysiological mechanisms underlying PTSD remain poorly understood despite the numerous experimental studies carried out over the last 30 years. Although the vast majority of the research in the field is focused on noradrenergic mechanisms (Naegeli et al., 2018), growing evidence suggests that other neural systems may be involved in the etiology and pathophysiology of PTSD. Great attention in this respect has been given to the endocannabinoid system. This neuromodulatory system is altered in PTSD patients (Hauer et al., 2013; Hill et al., 2013, 2018) and represents a potential therapeutic target in the treatment of PTSD (Fidelman et al., 2018; Morena et al., 2018). Intriguingly, a valuable body of findings demonstrate that the dopaminergic (DAergic) system controls behavioral responses to stressful situations (Pruessner et al., 2004). Various coping strategies to stressful events are sustained by fluctuations of tonic dopamine (DA) levels in the nucleus accumbens (NAc) (Cabib and Puglisi-Allegra, 2012) and manipulations of midbrain DAergic transmission alter resilience to stress (Friedman et al., 2014). A prominent role of DA into the prefrontal cortex (PFC) in mediating responses to stressful events has been also proposed (Pascucci et al., 2007) and, more interestingly, increasing evidence suggests that DAergic dysfunction might play a pivotal role in PTSD (Table 1). In the present review, DAergic mechanisms relevant for the pathogenesis of PTSD, as well as potential DAergic-based pharmacotherapies are discussed in the context of addressing the unmet medical need for new and effective drugs for treatment of PTSD.

**Table 1:** Findings from human studies demonstrating an impact of DAergic dysfunction on PTSD.

Dopaminergic dysfunction	Description	References
↑ Urinary DA levels	↑ Severity of PTSD symptoms (especially intrusion symptoms) CRITERION B	Yehuda et al., 1992
VNTR DAT SLC6A3 3′ polymorphism	Excess of nine repeat allele in PTSD patients	Segman et al., 2002
VNTR DRD4 exon III polymorphism	↑ Severity of PTSD symptoms on the Avoidance/Numbing scale. CRITERION C	Dragan and Oniszczenko, 2009
VNTR DAT SLC6A3 3′ polymorphism	↑ Arousal symptoms CRITERION E	Drury et al., 2009
COMT Val158Met polymorphism	↑ Risk of PTSD in Met/Met homozygotes	Kolassa et al., 2010
COMT Val158Met polymorphism	Attenuation of the effect of PTSD-related processes on right anterior cingulate cortex volume	Schulz-Heik et al., 2011
↑ DAT density	↑ Striatal DAT density in PTSD patients	Hoexter et al., 2012
COMT Val158Met polymorphism	↑ Fear to safety signal and impaired fear extinction in PTSD Met/Met homozygotes as compared to Val allele carrier CRITERION B	Norrholm et al., 2013
DRD3 single nucleotide polymorphisms (rs2134655, rs201252087, rs4646996, and rs9868039)	The minor alleles give resilience against PTSD	Wolf et al., 2014
COMT Val158Met polymorphism	Smaller left hippocampus in PTSD Val/Val homozygotes as compared to Met allele carriers	Hayes et al., 2017
COMT Val158Met polymorphism	Childhood trauma-dependent association between the Met/Met genotype and fear inhibition/extinction deficit CRITERION B	Deslauriers et al., 2018
COMT Val158Met polymorphism	Better working memory and executive functions in PTSD Met carriers as compared to Val/Val homozygotes CRITERION D	Havelka Mestrovic et al., 2018
		


## Daergic System

In the late 1950s, Arvid Carlsson discovered that the catecholamine DA was not just the precursor of norepinephrine (NE) but also a neurotransmitter, *per se* (Carlsson, 1959). DA exerts profound influences over several physiological functions including reward, cognition and emotional processes through the activation of two classes of DA receptors, the D1-like receptors (D1R and D5R) and the D2-like receptors (D2R, D3R, and D4R), which are G-protein-coupled receptors coupled to G_s_ and G_i_ protein, respectively (Leggio et al., 2016). DA is synthesized and released by DAergic nuclei located in the midbrain, whose projections create four major DAergic pathways (Hillarp et al., 1966). DAergic neurons located in the ventral tegmental area (VTA) send projections mainly to the PFC (mesocortical pathway) and the ventral striatum (mesolimbic pathway). The nigrostriatal pathway consists of DAergic projections from the substantia nigra to the dorsal striatum. Lastly, the DAergic neurons of the tuberoinfundibular pathway originate in the arcuate nucleus of the hypothalamus and terminate in the median eminence. Besides these classic DAergic nuclei, relatively less-studied DAergic neurons in the ventral periaqueductal gray (vPAG) and dorsal raphe nucleus (DRN) finely modulate specific physiological functions, such as associative learning of fear, arousal states and social behavior (Matthews et al., 2016; Cho et al., 2017; Groessl et al., 2018), which are altered in several neuropsychiatric disorders including PTSD. In line with these observations, converging lines of evidence indicate that DAergic dysfunction may subserve the development of the pathophysiological processes underlying several symptoms of this disorder.

## Da and Intrusion Symptoms

**FIGURE 1:**
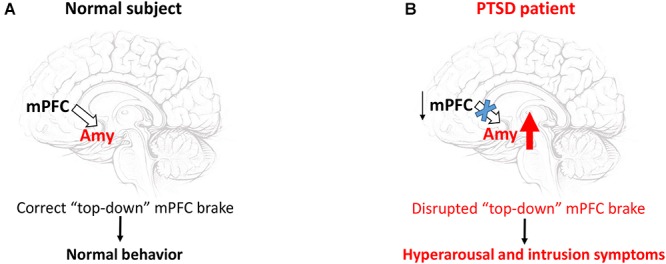
The failure of the “top-down” inhibitory control exerted by the mPFC over the amygdala triggers hyperarousal and intrusion symptoms. In a normal subject **(A)**, the mPFC exerts a homeostatic inhibitory control over the amygdala (Amy). This “top-down” brake maintains appropriate behavior patterns under stressful conditions. In PTSD patients **(B)** the hypoactive mPFC removes the brake over the Amy, whose hyperactivity mainly generates hyperarousal and intrusion symptoms (Fenster et al., 2018, see also text).

Criterion B of the DSM-5 criteria for PTSD diagnosis refers to intrusion symptoms. Intrusive re-experiencing of traumatic events represents a hallmark symptom of PTSD. This re-experiencing may occur in several ways such as flashbacks, repetitive intrusive memories and distressing dreams (Brewin, 2015). Growing evidence indicates that a failure of the top-down inhibitory control exerted by the medial prefrontal cortex (mPFC) over the limbic regions and especially over the amygdala is responsible for these intrusion symptoms (Figure 1; Lanius et al., 2010). Particularly, the impairment of cortical inhibitory control seems to be intimately related to a disrupted fear extinction (Milad et al., 2009), which contributes to exaggerated fear associated with the recurrence of traumatic memories. In the PFC, DA plays a pivotal role in regulating extinction memory consolidation (Hikind and Maroun, 2008; Mueller et al., 2010; Haaker et al., 2013; Gerlicher et al., 2018). Indeed, the pharmacological blockade of the D1R in the infralimbic cortex (IL) induced conditioned fear responses through a disruption of extinction consolidation in rats trained for auditory fear conditioning (Hikind and Maroun, 2008). On the other hand, a dietary zinc restriction-induced long-term rescue of deficient fear extinction has been associated with an enhancement of DAergic D1R and D2R receptor gene expression in the mPFC of a particular strain (129S1/SvImJ) of mice, which are basically characterized by deficient fear extinction acquisition and impaired fear extinction consolidation/retrieval (Whittle et al., 2016). This view is further substantiated by recent data showing that L-DOPA-induced burst of DAergic activity during extinction consolidation ameliorated extinction memory retrieval by increasing spontaneous ventromedial prefrontal cortex (vmPFC) reactivations (Gerlicher et al., 2018). The vmPFC has reciprocal connections with a key brain region involved in the acquisition and expression of conditioned fear, namely the amygdala (Phelps et al., 2004; Stevens et al., 2013). Hitora-Imamura et al. (2015) demonstrated a mesocortical DA-dependent modulation of fear extinction involving the interconnection between the IL and the central nucleus of the amygdala (CeA) (Hitora-Imamura et al., 2015). The shock reminder-induced release of DA within the IL dampens the activity of this cortical region, which in turn removes the “brake” on the CeA. This aberrant mechanism triggers the fear reinstatement. Among the amygdaloid nuclei, the CeA receives the most abundant DAergic fibers from the mesolimbic DA-containing neurons (de la Mora et al., 2010). These fibers release DA within the CeA under fear responses (Naylor et al., 2010). Notably, the DAergic vPAG/DRN neurons also represent a great source of DAergic fibers projecting to the CeA (Matthews et al., 2016). This DAergic vPAG/DRN – CeA circuitry has been recently proposed as a new pivotal participant of the Pavlovian fear conditioning processes. It couples a positive aversive prediction error signal (a central component of reinforcement learning, representing the mismatch between the actual outcome and the predicted outcome) to a DAergic reinforcement of an experience-dependent fear memory trace into the CeA (Groessl et al., 2018). Thus, a dysfunction of the DAergic vPAG/DRN – CeA circuitry together with a DA-dependent failure of the top-down inhibitory control exerted by the mPFC primarily over the hyperactive CeA might be responsible for the aberrant fear memory formation in PTSD patients.

At a clinical level, the severity of PTSD symptoms, particularly that of the intrusion symptoms, has been correlated to augmented urinary DA and NE levels (Yehuda et al., 1992). An inverse relationship between plasma DA and heart rate during fear inhibition has been also found in military service members with more severe subthreshold PTSD symptoms (Costanzo et al., 2016). Other human studies have found the Catechol-O-Methyltransferase (COMT) Val158/108Met (rs4680) polymorphism associated with fear inhibition and extinction deficits in PTSD. COMT is an enzyme that catalyzes the O-methylation and thereby the inactivation of catecholamines. Met/Met carriers exhibit a 40% reduction in COMT activity that leads to a heightened DAergic tone, especially in the cortex (Chen et al., 2004). In general, either enhanced fear memory consolidation or fear extinction resistance has been reported in Met/Met carriers (Lonsdorf et al., 2009, 2010). In a fear-potentiated startle study, PTSD patients with the Met/Met genotype showed higher fear to a safety signal as well as a more impaired fear extinction as compared to those patients carrying the Val allele (Norrholm et al., 2013). These findings have been further confirmed in a more recent study in which the authors discovered a childhood trauma-dependent association between the Met/Met genotype and a fear inhibition/extinction deficit (Deslauriers et al., 2018). Overall, these preclinical and clinical results indicate that DAergic mechanisms/dysfunctions may be deeply correlated to intrusion symptoms.

## Da and Avoidance Symptoms

Criterion C of the DSM-5 criteria for PTSD diagnosis concerns attempting to avoid distressing, trauma-related stimuli, including thoughts, people, places, conversations, objects and more in general attempting to avoid emotional experiences. These symptoms are closely related to fear extinction deficits showed by PTSD patients. Indeed, greater avoidance symptomatology has been associated with greater activation of fear circuits in a sample of veterans with PTSD (Sripada et al., 2013). In this respect, Stein and Paulus hypothesized that PTSD would represent a disorder originating from an altered homeostatic balance between the approach system and the avoidance system (Stein and Paulus, 2009). By definition, approach indicates the tendency to move toward positively valenced stimuli and it is closely linked to reward processes. Conversely, avoidance is the tendency to turn away from negatively valenced stimuli and, as mentioned before, is more connected to fear-related mechanisms. DA is the most important neurotransmitter regulating the reward-related learning of approach behavior (Contreras-Vidal and Schultz, 1999; Schultz, 1999, 2007). Moreover, animal studies demonstrate a key involvement of DA in avoidance behavior. DA in the PFC modulates top-down circuits involved in encoding aversive stimuli, able to trigger avoidance and escape-related defensive behaviors (Vander Weele et al., 2018). However, a more important role is played by subcortical DAergic mechanisms. Indeed, the photo-stimulation of DAergic neurons in the midbrain enhances active avoidance behavior and accelerates the extinction of passive avoidance behavior (e.g., freezing) under the presentation of a fear-conditioned cue. By contrast, the photo-inhibition of the same neurons, as well as the accumbal D1R antagonism, attenuate the active avoidance behavior (Wenzel et al., 2018). Worthy of note, optogenetic activation of the aforementioned DRN DAergic neurons increases place avoidance (Matthews et al., 2016).

A fundamental role of striatal DA in mediating avoidance behavior is also documented. Genetic deletion of the D2R on striatopallidal neurons increases avoidance behavior (LeBlanc et al., 2018) and activation of DAergic axons innervating the posterior tail of the striatum reinforces avoidance of threatening stimuli (Menegas et al., 2018).

The human neurobiological basis of avoidance symptoms has been less studied in PTSD. With regard to DA, one human genetic study has reported a significant association between the polymorphism of DRD4 exon III and the severity of PTSD symptoms on the Avoidance/Numbing scale (Dragan and Oniszczenko, 2009).

## Da and Alteration in Mood and Cognition

Criterion D of the DSM-5 criteria for PTSD diagnosis refers to the persistent negative changes in mood and cognition. PTSD patients may indeed experience cognitive dysfunctions such as dissociative amnesia (inability to recall crucial features of the traumatic event), impairment of executive functions (working memory and cognitive flexibility), but also enduring negative emotions (horror, anger, guilt, and shame), loss of interest in previously rewarding activities, social detachment, and incapacity to experience positive emotions (anhedonia). In this respect, a mutual interaction between emotions and cognition has been observed in PTSD. In PTSD patients, trauma-related memories and trauma-related cues generate negative emotions that may interfere with ongoing cognitive processes (Hayes et al., 2012). For instance, a reward deficiency has been described in PTSD (Elman et al., 2005). Some PTSD patients are less motivated to obtain positive rewards and because the decision-making process is highly influenced by the motivational state, these individuals show a disrupted decision-making capacity (Hayes et al., 2012). The mesolimbic DAergic pathway is the key player in reward-seeking behavior (Pessiglione et al., 2006, 2007) and, as suggested by Enman et al. (2015), striatal DAergic dysfunction may contribute to the occurrence of PTSD symptomatology, including anhedonia and emotional numbing, by altering reward processes (Enman et al., 2015). In line with these findings, a clinical study reported that PTSD patients showed an increased striatal dopamine transporter (DAT) density in comparison with traumatizing asymptomatic individuals (Hoexter et al., 2012). Because reward processes mainly rely on DA activity in the ventral striatum, this greater DAT density may underlie DA-dependent aberrant reward system in PTSD patients. It is important to point out that anhedonia is also a characteristic and severe symptom of major depressive disorder (MDD) (Keedwell et al., 2005) and a high rate of comorbidity between PTSD and MDD has been observed (Flory and Yehuda, 2015). In this latter condition, reduced striatal DAT density seems to reflect a compensatory mechanism aimed at counteracting a chronic hypodopaminergic tone (Meyer et al., 2001). Conversely, as mentioned before, increased striatal DAT density in PTSD may be related to hyperdopaminergia (van de Giessen et al., 2009; Hoexter et al., 2012). Thus, it seems that opposite striatal DAergic abnormalities may generate anhedonia, through the disruption of the reward processing. In an attempt to reveal the neural substrates of this decision-making/reward connection in PTSD, Sailer et al. found that PTSD patients exhibited hypoactivation of both the NAc and PFC during the processing of gains in the late learning phase of a decision-making task (Sailer et al., 2008). Hence, cortical DAergic mechanisms may also be involved in the decision-making/reward connection, and, more in general, in cognitive dysfunctions characterizing PTSD. Veterans PTSD patients carrying the COMT Val158Met polymorphism and homozygous for the Val allele displayed a volume of the anterior cingulate cortex smaller than controls non-PTSD (Schulz-Heik et al., 2011). In the PFC, DAT is low expressed and does not contribute to DA reuptake; COMT plays a more substantial role, being responsible for approximately one half of the total DA clearance (Kaenmaki et al., 2010). Remarkably, a significant association between the COMT Val158/108Met polymorphism and working memory performance has been found in PTSD patients. PTSD met carriers showed better working memory and executive functions than Val/Val homozygotes (Havelka Mestrovic et al., 2018). Considering that the met allele is associated with reduced COMT activity, which leads to a slower prefrontal DA degradation (Chen et al., 2004), it may maintain adequate level of DA in the PFC, associated to better cognitive performance. The critical impact of the COMT Val158/108Met polymorphism on PTSD symptoms, particularly on PTSD cognitive dysfunctions, is supported by the observation that a cluster of Val/Val homozygotes with severe PTSD symptomatology showed smaller left hippocampus compared to those patients carrying the met allele (Hayes et al., 2017). The hippocampus is highly enriched in COMT (Matsumoto et al., 2003), therefore, reduced hippocampal DA availability in Val carriers may impair crucial processes for memory formation such as, neurogenesis and long term potentiation.

Although these findings suggest that mood and cognitive alterations in PTSD patients may be triggered by dysregulation of the DAergic system, additional research would be relevant to better clarify the impact of this DAergic dysfunction.

## Da and Hyperarousal Symptoms

**FIGURE 2:**
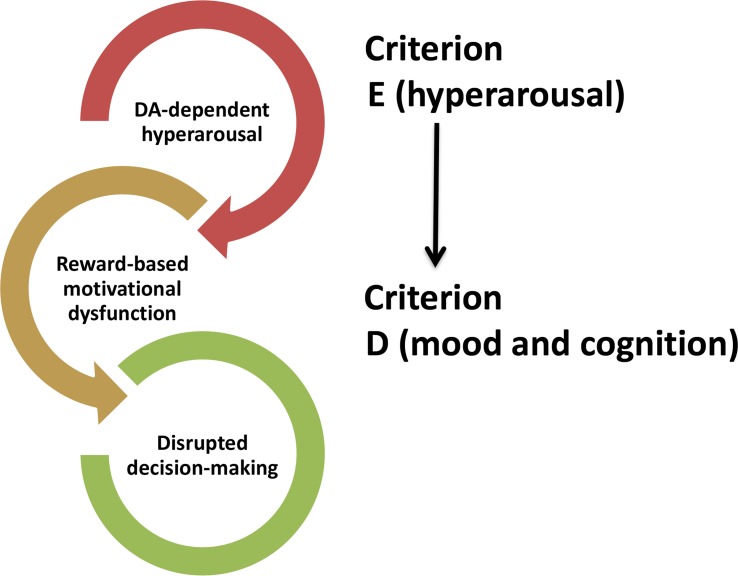
Hypothetical influence of DA-induced hyperarousal (CRITERION E) on symptoms (CRITERION D) occurring in post-traumatic stress disorder.

Criterion E of the DSM-5 criteria for PTSD diagnosis focuses on hyperarousal symptoms, including exaggerated startle, sleep disturbance, hypervigilance, impulsivity and aggressiveness. These symptoms often occur first (Bremner et al., 1996) and have a major impact in the natural course of the disease by influencing the development and onset of the other symptoms (Schell et al., 2004; Weston, 2014). Although NE is historically recognized as the key neurotransmitter in the modulation of arousal, DA is also involved in. DA fosters wakefulness (Monti and Monti, 2007) and VTA DAergic neurons play a central role in this effect. VTA DAergic neurons strongly fire during motivated waking (Dahan et al., 2007) and selective photo-stimulation of such DAergic neurons induces arousal on mice under isoflurane-induced continuous, steady-state general anesthesia (Taylor et al., 2016). Other studies further report a fluctuation of extracellular DA levels across different arousal states in the NAc and mPFC (Lena et al., 2005), the two main brain regions receiving DAergic projections from the VTA. However, VTA DAergic inputs to the NAc but not to the mPFC have the most fundamental role in mediating arousal states and ethologically relevant sleep-wake behaviors (Eban-Rothschild et al., 2016). VTA DAergic neurons are not the only DAergic neurons regulating arousal states. Lu et al. (2006) were able to label the vPAG DAergic neurons as wake-active DAergic neurons (Lu et al., 2006), showing that they project to several brain regions belonging to the wake–sleep regulatory system, including the mPFC. Thus, DA fluctuations within the mPFC (Lena et al., 2005) across different arousal states are likely triggered by vPAG DAergic neurons. A more recent study reveals that DRN DAergic neurons tune arousal and promote wakefulness upon the presentation of salient stimuli (Cho et al., 2017). All these observations prompt us to hypothesize that dysfunctional alterations of the midbrain DAergic neurons and their respective DAergic circuitries might underlie the sleep disturbances of PTSD patients, which may also explain the high contribution of hyperarousal symptoms on the occurrence of the other PTSD symptoms. For instance, a DA-dependent hyperarousal state could interfere with motivational processes, which are known to influence cognitive processes such as decision-making (Figure 2).

Relatively few studies have explored the role of DA in the startle reactivity. An increase of the DAergic signaling obtained by systemic administration of DA agonists is able to strengthen the startle reactivity (Davis et al., 1986; Meloni and Davis, 2000). Conversely, the genetic deletion of D2R and D3R produces an increase of startle habituation as well as a decrease of startle reactivity (Halberstadt and Geyer, 2009; Torrisi et al., 2017). These preclinical findings are substantiated by human studies reporting a connection between polymorphisms of genes implicated in DA metabolism and variable startle reactivity. The COMT Val158/108Met polymorphism is one of the polymorphisms affecting the startle reactivity. In a human genetic study, COMT Val carriers exhibited a heightened unpleasant stimuli-induced startle that was not modified by L-DOPA administration, while COMT Met/Met carriers did not show any change in startle under the presentation of unpleasant stimuli or neutral stimuli, but they displayed heightened unpleasant stimuli-induced startle after L- DOPA administration (Domschke et al., 2015).

Aggressive and/or dysfunctional impulsive behavior elicited by perceived threat is another common hyperarousal symptom characterizing PTSD. Aggressive and/or dysfunctional impulsive behavior has been linked to a deficient cortical brake over the limbic regions similarly to the disrupted fear extinction discussed above (Figure 1; Fenster et al., 2018). DA is also believed to be a key modulator of impulsive behavior (Dalley and Roiser, 2012; Besson et al., 2013). Intriguingly, a low prefrontal DAergic tone has been associated with high impulsivity, while increasing cortical levels of DA diminishes impulsive choice by varying corticostriatal connectivity (Kayser et al., 2012). Other studies provide clear evidence of a prominent contribution of subcortical DAergic mechanisms in aggressive and/or impulsive behavior. Optogenetic activation of VTA DAergic neurons raises aggressive behavior (Yu et al., 2014) and substantia nigra/VTA D2/D3 autoreceptor availability inversely and positively correlates with impulsivity and amphetamine-induced DA release in the striatum, respectively (Buckholtz et al., 2010). Hence, DAergic dysfunction might have a critical role in the development of aggressive and/or dysfunctional impulsive behavior exhibited by PTSD patients.

To our knowledge, only one clinical study (Drury et al., 2009) has provided direct evidence that DAergic dysfunction has a major influence on hyperarousal symptoms compared to other PTSD symptoms. Again, genetically induced variations of the DAergic system have a critical role in this regard. Genetic variants of the variable number tandem repeat element of 40 base pairs located in the 3′ UTR of the DAT gene have been related to variable transcription rate (Heinz et al., 2000; Michelhaugh et al., 2001). The nine repeat allele, whose excess has been found in a cluster of PTSD patients (Segman et al., 2002), has been specifically associated with hyperarousal symptoms (Drury et al., 2009).

## The Potential Therapeutic Value of Targeting the Daergic System in PTSD

The human and animal data discussed in this review suggest that DAergic dysfunction is implicated in the pathophysiology of PTSD and, as a consequence, drugs targeting the central DAergic system could have a therapeutic value for the management of this disorder. As mentioned above, the failure of the top-down inhibitory control exerted by the PFC over the limbic regions might be one of the key aberrant mechanisms subserving predominantly both the intrusion and the hyperarousal symptoms (Fenster et al., 2018). Because the reactivation of the PFC may restore the homeostatic inhibitory control over the subcortical regions and promote the consolidation of fear extinction, which is believed to subserve resilience against PTSD (Gerlicher et al., 2018), an effective strategy might be the administration of drugs known to boost the activity of this region via increasing the DAergic signaling. In this regard, a promising candidate drug might be methylphenidate, a NET and DAT blocker. Low doses of methylphenidate preferentially increase prefrontal catecholaminergic neurotransmission (Berridge et al., 2006). It dose-dependently increases extinction of contextual fear (Abraham et al., 2012) and, most importantly, has been shown to be effective on veterans suffering from PTSD and largely refractory to standard medications (Houlihan, 2011). As discussed above, L-DOPA increases spontaneous vmPFC reactivation via strengthening the DAergic signaling. In an ongoing clinical trial (NCT02560389), the investigators are testing the hypothesis that this commercially available drug may enhance fear extinction learning in PTSD women patients. In this respect, we speculate that a combination of these DAergic drugs able to promote the consolidation of fear extinction with CBT could be a successful strategy that can significantly diminish the relapse of PTSD symptoms. However, it is important to state that such drugs also affect the DAergic signaling in the subcortical regions and this effect may create issues because abrupt variation of the subcortical DAergic tone may facilitate the development of drug abuse (Volkow, 2006). One intriguing drug endowed with preferential activity on the PFC is the atypical stimulant atomoxetine. This drug, which is a potent and selective blocker of NE re-uptake approved for the treatment of attention-deficit/hyperactivity disorder, selectively increases DA levels in the PFC without affecting the subcortical DAergic system (Bymaster et al., 2002). This peculiar effect is based on the fact that, in the PFC, the re-uptake of DA is carried out by the highly expressed NET instead of the low expressed DAT (Yamamoto and Novotney, 1998). Several studies demonstrate that atomoxetine is able to ameliorate impulsivity and executive function deficits, which are found across different neuropsychiatric disorders (Ansquer et al., 2014; Borchert et al., 2016). Thus, atomoxetine might be effective in PTSD patients mainly showing hyperarousal symptoms. COMT inhibitors are another class of compounds that could restore the proper functioning of the PFC by increasing the DAergic signaling. As extensively discussed above, COMT Val158/108Met polymorphism has been associated with PTSD. The Val allele, being linked to higher COMT activity, lowers synaptic DA availability and disrupts PFC function (Chen et al., 2004). Therefore, we speculate that COMT inhibitors could be effective on Val/Val homozygotes PTSD patients. It is important to remark that PFC presents a high sensitiveness to catecholamine levels (Cools and D’Esposito, 2011). Small changes of NE and DA levels can markedly affect PFC-dependent brain function. It is thus important to select the correct dose of a given DAergic drug to reach the optimal DA levels, adequate for the PFC.

It has been further suggested that D3R antagonists may be effective cognitive enhancers in individuals with neuropsychiatric disorders (Nakajima et al., 2013). The pro-cognitive effect of D3R antagonists has been linked to a facilitation of PFC top-down control of subcortical brain regions, implicated in the modulation of cognition (Millan et al., 2007; Loiseau and Millan, 2009). To date, only two studies have examined the interaction between D3R and PTSD.

In a candidate gene study, the minor alleles of four DRD3 single nucleotide polymorphisms (rs2134655, rs201252087, rs4646996, and rs9868039) have been found to be protective against the risk for PTSD (Wolf et al., 2014). Preclinical findings have also demonstrated that the selective blockade of D3R ameliorates the PTSD-like behavior of rats, previously exposed to the single-prolonged stress model (Song et al., 2018). These observations indicate that the D3R may be considered an interesting pharmacological target for the development of new effective drugs for the treatment of PTSD. From another perspective, commercially available drugs targeting the D3R might undergo a drug repositioning process. This process aims to discover new uses for already approved and commercialized medications and it offers great advantages over the long-lasting, risky and expensive, *de novo* drug discovery. In this context, two intriguing commercially available drugs are the antipsychotic aripiprazole and cariprazine, both endowed with D2R/D3R partial agonist activity and preferential binding to D3R (Leggio et al., 2016). By definition, partial agonists may act either as agonist or antagonist according to the surrounding levels of the neurotransmitter. Thus, these drugs may result effective on a wide range of PTSD patients characterized by a condition of DA imbalance, triggered by different genetic and epigenetic factors. This view is substantiated by a recent study showing that aripiprazole improved fear extinction retrieval via a normalization of the DA efflux in the amygdala (Lin et al., 2019). Finally, as mentioned above, ketamine, a drug candidate for PTSD, enhances DAergic neurotransmission in both humans and rodents (Lindefors et al., 1997; Kegeles et al., 2000; Can et al., 2016) and its activity on DAergic neurons relies on functional intact D3R (Cavalleri et al., 2018).

## Conclusion

Currently available pharmacotherapies for PTSD are poorly effective on a substantial proportion of patients. Given this high rate of pharmacological unresponsiveness, further studies are needed to extend the knowledge of the basic mechanisms associated with the pathophysiology of this disorder. The findings discussed in this review suggest that DAergic dysfunction, especially genetic-dependent DAergic alteration, plays a prominent role in the pathophysiology of PTSD; as a consequence, drugs targeting the DAergic system might be therapeutically relevant. A better understanding of how and which DAergic dysfunction contributes to the symptoms of PTSD patients with different genetic background may lead to the development of effective drugs and more personalized treatments.

## Author Contributions

All authors listed have contributed to the work, by searching the literature, critical reading, and summarizing published data and writing this review. All authors listed have approved this review for publication.

## Conflict of Interest Statement

The authors declare that the research was conducted in the absence of any commercial or financial relationships that could be construed as a potential conflict of interest.
